# CardioMEMS in a Busy Cardiology Practice: Less than Optimal Implementation of a Valuable Tool to Reduce Heart Failure Readmissions

**DOI:** 10.1155/2018/4918757

**Published:** 2018-11-07

**Authors:** Robby Singh, Leon Varjabedian, Georgy Kaspar, Marcel Zughaib

**Affiliations:** Providence Hospital, Michigan State University, Detroit, MI, USA

## Abstract

**Introduction:**

Congestive heart failure is a leading cause of cardiovascular morbidity and mortality that results in a significant financial burden on healthcare expenditure. Though various strategies have been employed to reduce hospital readmissions, one valuable tool that remains greatly underutilized is the CardioMEMS (*Abbott*), a remote pulmonary artery pressure-monitoring system, which has been shown to help reduce heart failure rehospitalizations in the CHAMPION (CardioMEMS Heart Sensor Allows Monitoring of Pressure to Improve Outcomes in NYHA Class III Heart Failure Patients) trial.

**Methods:**

ICD-9/ICD-10 codes for chronic heart failure were used to identify patients who presented with congestive heart failure. Of this group, those eligible for CardioMEMS device placement, as based on the CHAMPION trial definition, were selected. Subsequently, a retrospective review of the electronic medical records was completed. All patients were on ACC/AHA guideline-directed medical therapy and had at least one hospital admission for NYHA class III symptoms.

**Results:**

473 patients met the inclusion criteria, of which, 85 patients were found to be eligible for implantation of CardioMEMS device based on the CHAMPION trial definition. Only 18/85 patients received the device, roughly 21%, and the overall CardioMEMS implantation rate was only 4% (18/473) of the total cohort.

**Conclusion:**

Despite the benefits to patients and reducing healthcare expenditure, there has been a poor adaptation of this groundbreaking technology. Our study revealed that 79% of eligible heart failure patients did not receive the device. Therefore, efforts need to be undertaken to improve physician and patient education of the device to complement the current standard of care for congestive heart failure.

## 1. Introduction

Congestive heart failure is a leading cause of cardiovascular morbidity and mortality in the US and results in a significant financial burden on healthcare expenditure, particularly with hospital readmissions. While various strategies have been employed to reduce readmissions, such as remote monitoring of patient weights, early cardiology consultations in the emergency room, and prompt outpatient follow-up after discharge, one valuable tool that remains greatly underutilized is the CardioMEMS (*Abbott*), a remote pulmonary artery pressure-monitoring system.

High cardiac filling pressures in patients with heart failure are associated with a higher risk of hospitalization and mortality, regardless of left ventricular ejection fraction. A retroactive subgroup analysis of the COMPASS-HF (Chronicle Offers Management to Patients with Advanced Signs and Symptoms of Heart Failure) study [1] showed that an active adjustment in medications in response to elevated filling pressures transmitted by an implanted device significantly decreased hospitalizations in NYHA class III patients [2]. In patients with heart failure, decreasing the pulmonary artery diastolic pressure to less than 25 mm Hg decreased heart failure events by 50% [1]. In the CHAMPION (CardioMEMS Heart Sensor Allows Monitoring of Pressure to Improve Outcomes in NYHA Class III Heart Failure Patients) trial, pulmonary artery pressure-guided heart failure management was associated with 28% reduction in heart failure hospitalization after 6 months and 37% after 15 months compared with clinical assessment-guided therapy alone [3].

CardioMEMS is an FDA-approved device that has been shown to significantly reduce heart failure hospital admission and improve the quality of life in New York Heart Association (NYHA) class III patients [3] by remote monitoring of pulmonary artery pressures, an indirect measurement of left ventricle volume load. Recent studies have shown a significant improvement in exercise capacity, in addition to the quality of life, in patients managed with a remote pulmonary artery pressure-monitoring device compared with the standard of care heart failure management [4]. This technology, when implanted in the appropriate clinical setting, can significantly reduce unnecessary admissions related to acute heart failure exacerbation in patients with either heart failure with preserved or reduced ejection fraction. Costanzo et al. [5] found that remote monitoring of the pulmonary artery pressure data for actively managing heart failure in all patients by targeting lower filling pressures led to increased use of diuretics, vasodilators, and neurohormonal antagonists and overall more frequent medication adjustments and decreased heart failure hospitalizations. A similar outcome was also found in patients with heart failure and reduced ejection fraction [6].

In addition, these findings were not limited only to clinical trials, but were also seen in real-world settings. Heywood et al. [7] found that the first 2000 general practice use patients who underwent remote pulmonary artery pressure monitoring had a greater reduction in PA pressure than the patients in the CHAMPION trial. Similarly, Desai et al. [8] found that the use of remote pulmonary artery pressure monitoring was also associated with lower heart failure hospitalizations and decreased total heart failure costs in a real-world setting. However, in cardiology practices across the United States, there remains an unmet need for wider adoption of this breakthrough technology, resulting in a potentially negative impact on patient management. In this study, we sought to determine the extent of this unmet need and to postulate potential explanations for it with a discussion of opportunities for improvement.

## 2. Objective

The objective of this study was to identify patients that would meet the inclusion criteria for the CHAMPION trial and qualify for the implantation of a CardioMEMS device for pulmonary artery pressure monitoring, such as patients with NYHA class III symptoms and at least one hospital admission in the past 12 months. The goal of this study was to determine the extent of the unmet need for this technology that has been shown to reduce hospital readmissions for heart failure patients regardless of their LV systolic function and therefore reduce healthcare costs, in addition to the associated morbidity of recurrent heart failure admissions.

## 3. Methods

**Figure 1:**
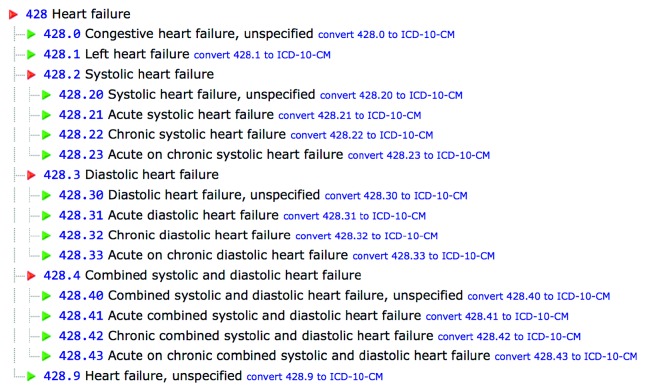
ICD-9 codes for heart failure.

**Figure 2:**
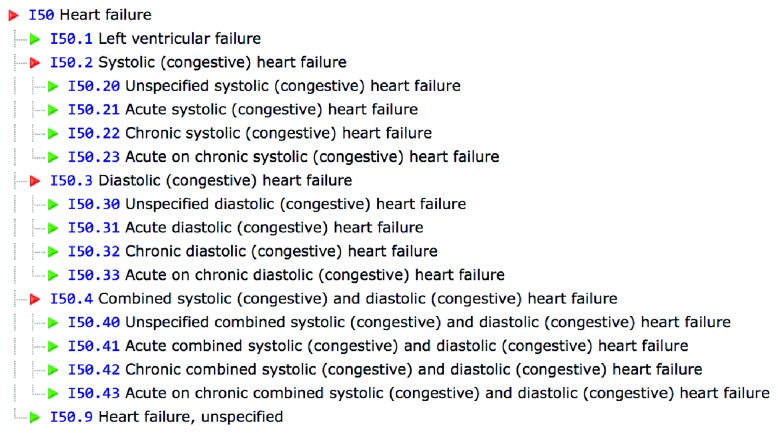
ICD-10 codes for heart failure.

ICD-9/ICD-10 codes (Figures 1 and 2) for chronic heart failure were used to select patients from a busy hospital-associated outpatient cardiology practice, *Heart Cardiology Consultants P.C.*, in southeast Michigan, which comprises 13 cardiologists with academic appointments and teaching responsibilities combined with private practice. A retrospective review of the clinic electronic medical records (EMR), Aprima®, selected patients between January 2015 and February 2016 clinic visits. Heart failure patient selection was irrespective of whether the patient had heart failure with reduced or preserved ejection fraction, ischemic cardiomyopathy, or nonischemic cardiomyopathy and used the New York Heart Association classification system in accordance with patients' reported symptoms. All patients were on ACC/AHA guideline-directed medical therapy. Once the patients were deemed to be candidates for remote pulmonary artery pressure monitoring, the device was implanted by an interventional cardiologist. This was a retrospective study, and the EMR review was compliant with HIPAA regulations and aimed to identify at least one hospital admission for NYHA class III symptoms and determine if the patients received the remote pulmonary artery pressure-monitoring device.

## 4. Results

**Figure 3:**
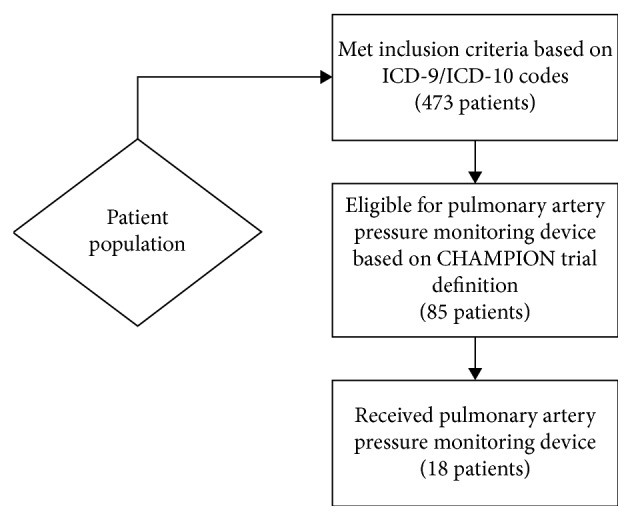
Of the 473 patients with CHF, 85 patients were eligible for remote pulmonary artery pressure monitoring though only 18 patients received the device.

Four hundred and seventy three patients met the inclusion criteria based on the ICD-9/ICD-10 codes for CHF during the defined period of time, of which, 85 patients were found to be eligible for implantation of CardioMEMS device based on the CHAMPION trial definition. All of the eligible patients were on guideline-directed medical therapy for either heart failure with reduced ejection fraction (HFrEF) or heart failure with preserved ejection fraction (HFpEF). The CardioMEMS eligibility ratio, the ratio of patients eligible for device placement to the total number of patients in the heart failure cohort, was 18% (85 out of 473 patients who met the inclusion criteria). Surprisingly, only 18 patients received the device out of the 85 patients eligible for it, roughly 21%, and the overall CardioMEMS implantation rate was only 4% of the total cohort, since only 18 of the 473 patients who met the inclusion criteria for CHF readmissions underwent device placement (Table 1, Figure 3). A total of 3 interventional cardiologists implanted the device across the 18 patients who followed up with their own cardiologists in the practice. Roughly 40% of the patients who underwent pulmonary artery pressure monitoring were males, while 60% were females, and the average age at implantation was roughly 75 years old, in contrast to the CHAMPION trial in which the mean age was 60 years old with 75% of the patients being male in the treatment group [6].

## 5. Discussion

Our study reveals that, in a busy outpatient cardiology practice, 79% of heart failure patients who were eligible for and would have benefited from remote pulmonary artery pressure monitoring did not receive the device. There are several traditional methods of monitoring physiologic parameters of CHF patients such as changes in patients' weight, symptoms, thoracic impedance, and heart rate variations on telemetry that can help determine the risk of CHF exacerbation hospital admission. The Tele-HF trial [9] showed that among 1,630 patients recently hospitalized for heart failure, telemonitoring of subjective symptoms did not reduce all cause readmissions death and heart failure readmissions, a result further supported by the TIM-HF trial [10], which also included noninvasive data such as ECG telemetry.

Similarly, measuring intrathoracic impedance by means of applying a subthreshold electrical impulse between the tip of the right ventricular lead and the generator in patients with implantable cardioverter-defibrillators or cardiac resynchronization therapy is another indirect method of measuring pulmonary fluid content, hence left ventricular filling pressure. A decline in thoracic impedance, which reflects pulmonary capillary wedge pressure, occurred 10 to 15 days before the clinical onset of heart failure symptoms in patients with NYHA III and IV CHF [11]; however, this also did not result in decreased heart failure admissions in the DOT-HF trial [12]. All of these indices lack the daily, nearly real time, data a remote pulmonary artery pressure-monitoring system, such as the CardioMEMS device, provides [13]. The proven usefulness of the CardioMEMS device is seen with improved patient outcomes [3] and presumed improvement in mortality and healthcare savings.

Roughly, 3.4 million encounters for outpatient care of heart failure add to the healthcare cost burden, while roughly one quarter of discharged patients are readmitted within 30 days [14, 15]. The CHAMPION trial showed not only an improvement in the quality of life but also a reduction in heart failure hospital admissions and a decreased length of admission [3]. Furthermore, there was a $7,433 six-month comprehensive heart failure cost reduction associated with the lower rate of hospitalizations [16], and the remote pulmonary artery pressure-monitoring device was also noted to have a cost-effectiveness ratio of $44,832 per quality-adjusted life year [17].

Despite the numerous benefits to patients and a positive impact on reducing healthcare expenditure, there has been a poor adoption of this groundbreaking technology, as evidenced in our study with only 21% of eligible patients receiving the device. There are a multitude of reasons that can help explain these findings. First and foremost, there are other methods available for remote monitoring of patients with CHF that continue to be used by many practitioners. There is a natural trepidation about placing a relatively expensive device in nonadherent patients as the device does require patient participation in data transmission. Additionally, there may be a hesitation on the physicians' part to solely treat a number. The foundation of clinical practice and patient care is based on a thorough history and physical exam, a concept engraved in the minds of all physicians early on in training. However, the idea of managing patient care using solely the remote pulmonary artery pressure-monitoring device is erroneous; the ability to monitor pulmonary artery pressures remotely is adjunct to the current methods of managing CHF patients and is not meant to replace good clinical judgement and care, but rather, enhance it.

Although there may be several reasons for the low utilization of remote pulmonary artery pressure-monitoring device, there are also many approaches to shift the current mindset of CHF management using remote pulmonary pressure monitoring. There needs to be a concentrated effort on increasing physician education and awareness of the benefits of remote pulmonary artery pressure monitoring in CHF patients. At the same time, patient education is also critical as patients should be aware of this new technology that can reduce emergency department visits and improve quality of life. It should start early by incorporating educational material into hospital discharge papers for all patients who qualify for remote monitoring at the time of their discharge as well as being made available as an adjunct to treatment for patients in the outpatient setting. It would also be beneficial to provide patients with educational pamphlets in the first outpatient visit after an admission for acute heart failure exacerbation to ensure appropriate education about the device. Though the process of adopting new concepts and technologies can be difficult and tedious, physicians have an obligation to their patients to be well informed of new treatment strategies for diseases and be willing to adapt to provide patients with the highest clinical care.

## 6. Future Directions

While educating physicians and the public is a key step in increasing the utilization of the CardioMEMS device in appropriate patients, it is also important to assess the larger impact of the device in patient care. In that regard, there are numerous large clinical trials that are either planned or underway to evaluate the added mortality benefits of remote pulmonary artery pressure monitoring, and more work needs to be done to fully assess the impact of remote pulmonary artery pressure monitoring in CHF patients. In addition, the GUIDE HF trial is underway and will evaluate the benefits of pulmonary artery pressure monitoring in NYHA class II and IV patients as well.

## 7. Conclusion

Congestive heart failure is a leading cause of cardiovascular morbidity and mortality and places an enormous burden on US healthcare expenditure, due in large part to readmissions for CHF exacerbation. CardioMEMS, a remote pulmonary artery pressure-monitoring device, has been shown to decrease CHF-related hospital readmissions and improve patients' quality of life. However, the device remains vastly underutilized in clinical practice, and efforts need to be undertaken to improve physician and patient education of the uses and benefits of the device to complement the current standard of care for congestive heart failure.

## Figures and Tables

**Table 1:** Total number of patients meeting inclusion criteria for CHF, total number eligible for CardioMEMS placement, and total number that received the device.

Total patients meeting CHF inclusion criteria for study	473	
Patients eligible for CardioMEMS device	85/473	18%
Number of eligible patients who received the device	18/85	21%
Number of patients who received the device out of the total cohort	18/473	4%

## Data Availability

The data used to support the findings of this study are available from the corresponding author upon request.
